# The impact of network positions in scientific collaboration on pharmaceutical firms' technological innovation performance: Moderating roles of scientific collaboration strength and patent stock

**DOI:** 10.3389/fpubh.2022.980845

**Published:** 2022-08-10

**Authors:** Xing-Xiu Wang, Hui-Ying Jiao

**Affiliations:** ^1^School of Management, Changchun University, Changchun, China; ^2^Jilin Provincial Key Laboratory of Human Health Status Identification and Function Enhancement, Changchun, China

**Keywords:** scientific collaboration, degree centrality, structural holes, scientific collaboration strength, patent stock

## Abstract

Scientific knowledge is an underlying basis for technological innovation in the pharmaceutical industry. Collaboration is the main way to participate in the creation of scientific knowledge for pharmaceutical firms. Will network positions in scientific collaboration affect their technological innovation performance? Moreover, what factors moderate the firms' scientific collaboration network positions and technological innovation link? Using a dataset based on 194 Chinese publicly traded pharmaceutical companies, this paper constructs the dynamic scientific collaboration networks among 1,826 organizations by analyzing 4,092 papers included in CNKI and Web of Science databases. Then we probe the impact and boundaries of positions in the scientific collaboration network of pharmaceutical firms on their technological innovation performance through the negative binomial modeling approach. Our study confirms that degree centrality has an inverted U-shaped impact on pharmaceutical firms' technological innovation performance, while structural holes benefit it. Moreover, this article identifies that the strength of scientific collaboration positively moderates the U-shaped relationship between degree centrality and technological innovation of pharmaceutical firms, the matching of high patent stock and high structural holes can promote their technological innovation performance. The results deepen the present understanding of scientific collaboration in the pharmaceutical industry and offer new insights into the formulation of pharmaceutical firms' scientific collaboration strategies.

## Introduction

In the era with a highly competitive and increasingly complex environment, a widely used strategy in the pharmaceutical industry is for firms to develop close linkages with universities, research institutes, and industries (URIs) (1). Scientific collaboration, mainly measured by scientific paper co-publication (2), is a common form of those linkages for pharmaceutical firms, where innovation is based on scientific advances (3). Take biopharmaceutical companies in the United States as examples, 116 biopharmaceutical companies published 7,000 papers between 1988 and 1994, among which 70% were published in collaboration with partners like universities and research institutes (4). Chinese pharmaceutical firms also actively participate in scientific collaboration, their co-published papers accounting for 80.76% of the total papers in our sample. Kafouros et al. (2) also noted that Chinese firms rely heavily on scientific collaborations due to their limited internal R&D capabilities. Thus, whether scientific collaboration enhances firms' innovation performance has received substantial interest (5–10). However, there is no agreement on this research topic in the existing literature (2). Some studies revealed that scientific collaboration can increase firms' capacity for problem-solving (2), foster interactive learning (9), and supply a pool of specialized labor (10), enhancing firms' innovation performance. In contrast, other studies argued that it also poses coordination and monitoring challenges due to the cognitive distance (11), divergent incentives, and different targets between firms and URIs (2, 12). To overcome the disagreement, Social Network Analysis (SNA) has been widely adopted in this field recently (3, 8, 13, 14).

From the social network perspective, pharmaceutical firms exchange information, ideas, knowledge, and resources with other actors in complex scientific collaboration networks constructed through scientific collaboration linkages (3). Extant literature has provided valuable insights indicating that actors' characteristics and network attributes were key explanations for pharmaceutical firms' innovation performance (5, 8, 15, 16). Specifically, the number of partners (15), collaboration diversity (5), network breadth, and network strength (8) were conductive to pharmaceutical firms' innovation performance. In addition, some studies investigated the moderating roles of scientific collaboration and firms' innovation links, such as the level of international openness (2), technological dynamics, and market dynamics (8). However, little literature has been focused on the impacts of pharmaceutical firms' network positions in scientific collaboration on their innovation performance and the moderating factors even though some studies have shown that network positions were beneficial to research institutes in their scientific collaboration (13). Meanwhile, the important impact of network positions in technical collaborative networks and strategic alliances on firms' innovation performance has long been the focus of many studies (17–19). Schilling and Phelps (17) proved that firms have greater innovative output when they are engaged in alliance networks of high reach and high clustering. Ahuja (18) posited that firms' direct ties and structural holes in collaboration networks were related to their subsequent innovation output. Wang et al. (19) indicated that network centrality positively influenced organizational innovation, and it was stronger for organizations in knowledge-intensive industries as well as in developed institutional environments. In addition, Yang et al. (8) pointed out that structural holes and network intensity still needed more work, as they were important dimensions of scientific collaboration networks. In this context, some valuable and important topics are worthy of our attention. Do network positions of pharmaceutical firms in scientific collaboration benefit their technological innovation performance? What factors moderate the link between scientific collaboration network positions and pharmaceutical firms' technological innovation?

To fill this gap, we examine how positions in scientific collaboration networks influence the technological innovation performance of pharmaceutical firms from the social network viewpoint. Integrating the framework of SNA and the Knowledge-based view (KBV), we argue that occupying key positions in scientific collaboration networks benefits pharmaceutical firms' technological innovation, and the value of such network positions depends on the strength of scientific collaboration and patent stock, respectively. We propose two typical network positions in scientific collaboration: degree centrality and structural holes, which play different roles in improving the technological innovation performance of pharmaceutical firms. *Degree centrality* has an inverted U-shaped association with technological innovation performance; however, *structural holes* positively enhance it. In addition, we suggest that the value of degree centrality and structural holes depends on scientific collaboration strength and patent stock, respectively: when the strength of scientific collaboration is high, the degree centrality may cause even better results; when the patent stock is richer, more structural holes exert superior and positive effects. To verify our hypotheses, we collected data on 194 Chinese publicly traded pharmaceutical companies during 2005–2021. The dataset involves the information of 4,092 publications by 1,826 organizations included in CNKI and Web of Science databases. The empirical results prove our research hypotheses.

Our study makes three contributions to the literature. First of all, compared with previous research, which mostly neglected firms' network positions in scientific collaboration and mainly focused on the impacts of network attributes and partner characteristics on innovation performance (8, 15), we investigate factors influencing the technological innovation performance of pharmaceutical enterprises in the perspective of network positions, which can offer some important empirical evidence for the influence of scientific collaboration on technological innovation. Furthermore, to provide a more detailed response to the question of how to enhance the value of various network positions in scientific collaboration networks, this research analyzes the moderating variables to the link between network positions and technological innovation performance. Lastly, this paper studies the inter-organizational scientific collaboration in China, enriching empirical research on scientific collaboration in the context of newly industrialized economies, which are fundamentally different from those of the developed countries (2).

## Theoretical background and hypotheses

### Scientific collaboration and innovation of pharmaceutical firms

The accumulation of scientific knowledge is a prerequisite for technological innovation in the pharmaceutical field. R&D in this industry is generally divided into three stages: government-funded basic research, publication of papers in pharmaceutical journals, and industry-led applied research (20). Research-intensive pharmaceutical firms can accelerate knowledge accumulation that integrates the knowledge of scientists inside and outside to generate valuable technological innovation (4). R&D activities of U.S. pharmaceutical firms increasingly emphasize the use of scientific knowledge generated by scientists at universities, particularly in bioscience-related fields, where inventions approved by the U.S. Food and Drug Administration are positively correlated with scientific publications (21). Sarkissian (22) argued that the top three factors positively influencing drug discovery were highly qualified R&D scientists, R&D investment, and excellent R&D management; the depth of specialized knowledge needed to be taken into account more than the diversity of knowledge available to the discovery teams; however, the main inhibitors to drug discovery were that clinical trials were challenging, the same drug targets were pursued, and drugs were designed with specialized or narrow therapeutic effects.

To date, different levels of research have been conducted on the link between scientific collaboration and innovation of pharmaceutical firms using SNA, including regional/country (3), organizational (2, 5, 8, 14, 15), and individual level (6, 23, 24). At the organizational level, relevant research was mostly focused on the impact of partner characteristics and network attributes in scientific collaboration on innovation performance. Mckelvey and Rake (15) built scientific collaboration networks based on co-publication papers by pharmaceutical firms and found that the number of partners, direct connections with academic institutions, and indirect linkages with academic institutions and biotechnology companies were conducive to pharmaceutical firms' product innovation, but their eigenvector centrality and betweenness centrality in the network had no impact on product innovation. Radicic and Pinto (16) proposed that collaboration between firms and universities and suppliers was conducive to product innovation and process innovation, and cooperation with suppliers was found to raise the tendency of innovation in industries with higher technology intensity; in contrast, collaboration with universities can increase the innovation possibilities in industries with lower technology intensity. Gao and Guan (14) displayed the characteristics of scientific collaboration networks from journals in six fields, e.g., Biotechnology, Pharmaceuticals, etc. to highlight differences in their network structures and identify heavily science-based networks. Lin (5) evaluated university-firm collaborations and found that knowledge stock, collaboration diversity, and collaboration ambition rather than the number of partnerships can lead to higher enterprise performance. In addition, some studies investigated the moderating roles of scientific collaboration and firms' innovation links. Kafouros et al. (2) proved the moderating roles of intellectual property rights enforcement, international openness level, and the research quality of URIs in the emerging market. Yang et al. (8) suggested that market and technological dynamics influenced how the breadth and strength of scientific collaboration networks affected company innovation.

Most of the previous studies recognized that scientific knowledge and scientific collaboration were important to the technological innovation performance of pharmaceutical firms, but mainly from the perspectives of partner's characteristics, network attributes, etc., only meager attention was paid to the relationship between network positions in the scientific collaboration and technological innovation of pharmaceutical firms, and there was also a lack of further discussion of its impact boundaries. In addition, most scientific collaboration networks were constructed from the co-publication papers in the Web of Science database (8). However, publishing papers in English is more difficult for Chinese pharmaceutical firms than publishing Chinese papers. Thus, co-publication in Chinese is the main way to participate in the scientific collaboration for pharmaceutical firms in China. In this context, only focusing on English publications cannot fully capture the actual situation of scientific collaboration networks in the newly industrialized economies. Therefore, we integrate social network theory and knowledge-based theory, take 194 publicly traded pharmaceutical companies as the research sample, and collect Chinese scientific papers published in the CNKI database, which were indexed by SCI, EI, PKU, CSSCI, and CSCD, and English scientific papers in Web of Science databases to build scientific collaboration networks. Using two crucial network measurements—namely, degree centrality and structural holes—we investigate the effects of network positions in scientific collaboration on the technological innovation performance of pharmaceutical companies. And we further explore the moderating role of the strength of scientific collaboration and patent stock on the link between network positions and technological innovation performance.

### Network positions and technological innovation performance of pharmaceutical firms

The scientific collaboration network describes the scientific collaboration relationship among various organizations or scientists using coauthored publications data, citation data, etc. (8, 24). In this study, scientific collaboration network refers to collaboration networks formed by pharmaceutical firms, universities, research institutes, hospitals, suppliers, etc. using co-authorship data, which indicate strong social connections (8). According to social network theory, network positions were measured by two typical indicators: degree centrality and structural holes (13).

### Degree centrality and pharmaceutical firms' technological innovation performance

Degree centrality refers to the direct links between pharmaceutical firms and other actors, which characterizes pharmaceutical enterprises' centralization in the scientific collaboration network (13, 14). The degree centrality of pharmaceutical firms will be higher if they have more direct links with other partners. In the scientific collaboration network, pharmaceutical firms' degree centrality is conducive to enhancing their technological innovation performance, however, it also comes at a cost. Specifically, degree centrality enhances technological innovation through various mechanisms. First, degree centrality is conducive to knowledge sharing which facilitates bringing together complementary skills from URIs. When all actors collaborate to create new scientific knowledge, the resultant knowledge is available to firms. Therefore, pharmaceutical firms can potentially receive a greater amount of knowledge from scientific collaboration networks compared to that from independent research investment (18). Pharmaceutical firms' partners in scientific collaboration networks are mainly URIs with complementary skills. Under such circumstances, degree centrality can enable firms to tap into the developed competencies of URIs to enhance their knowledge base and improve their innovation performance (25). Second, degree centrality enhances pharmaceutical firms' reputations. In social networks, a higher degree centrality represents a significant impact on partners, which means a great reputation (26). In the process of scientific collaboration, reputation is an important factor in attracting potential partners. With the help of reputation signals, pharmaceutical firms attract outstanding scientific and technological talents and improve their success rate of technological innovation (19). Third, it helps pharmaceutical firms acquire diverse information, a crucial factor to drive technological innovation. Compared to those in the peripheral position, pharmaceutical firms in the central position cover a wider range of the scientific collaboration network (27), and the higher degree centrality is conducive to rapid and effective access to a large amount of cutting-edge scientific information, which increases the scale of firms' technology information pool and avoids information asymmetry in the process of technological innovation (28), which in turn helps firms identify technological opportunities and improve the efficiency of technological innovation. The higher the degree centrality is, the more channels to obtain external information for firms, which can reduce the information search time, decrease the transaction costs between organizations, and avoid falling into path dependence on technological innovation. Mckelvey and Rake (15) also pointed out that the number of pharmaceutical firms' partners in the scientific network has a role in promoting product innovation performance. However, there exist two disadvantages when pharmaceutical firms occupy a high degree centrality in the scientific collaboration network. First, with an increase in the degree centrality, pharmaceutical firms have more ties in scientific collaboration networks, which brings large amounts of information and knowledge, undermining the timeliness and effectiveness of processing information and absorbing knowledge (13). It also may overload pharmaceutical firms, resulting in poor technological performance. Second, reaching and maintaining high centrality increases the cost of collaboration management. It also brings great challenges to firms' absorption capacity due to the cognitive distance between firms and URIs, so it is a great expense for pharmaceutical firms. If pharmaceutical firms fail to absorb the obtained scientific knowledge or integrate it into their existing technical knowledge, it leads to waste (19). Therefore, the technological innovation performance of pharmaceutical firms might be adversely affected by excessively high network centrality.

Given the advantages and shortages of degree centrality, we expect it will exert a nonlinear impact on pharmaceutical firms' technological innovation information. When pharmaceutical firms' degree centrality is at a low level, a higher value of degree centrality is beneficial to them because of more knowledge, high reputation, and diverse information, and enhances their technological innovation performance in the initial stage. However, when a certain point is reached, the benefits brought by degree centrality will be offset by its cost, and thus counterproductive results ensure. Therefore, the following hypothesis is proposed:

H1: In the scientific collaboration network, pharmaceutical firms' degree centrality has an inverted *U*-shaped effect on their technological innovation performance.

### Structural holes and pharmaceutical firms' technological innovation performance

Structural holes are used to describe such a network structure where two actors concurrently connect with a third actor but do not have direct links with one another, then the third actor acts as a broker (29). As non-redundant links between actors, structural holes can provide their occupants with opportunities to gain information and control benefits, thereby these actors are more competitive than members in other positions in the network. In the scientific collaboration network, pharmaceutical firms can gain three benefits from structural holes. The first is to access heterogeneous information timely. When pharmaceutical firms span more structural holes, which means they build more non-redundant and unique ties to link a large quantity of diverse URIs, they can obtain higher efficiency and more privileges to access heterogeneous information and resources in the network, for example, databases, facilities, and instrumentations (13). It may help pharmaceutical firms to integrate external heterogeneous resources into their innovation processes, so successively generate new ideas and new technologies. Structural holes are also conducive to identifying technological innovation opportunities in time by promoting the dissemination of scientific knowledge and realizing the inter-organizational transfer of scientific knowledge (18). The second benefit is to stimulate pharmaceutical firms' status accumulation. The occupants of structural holes act as brokers in the scientific collaboration network, bringing social capital to themselves (29) and enhancing their status (30). Yan and Guan (31) proposed that structural capital has positive effects on both exploitative and exploratory innovation. The third benefit is to control other nodes in the network and become the tertius gaudens. Structural holes holders have certain control over their partners in the scientific network, which will improve the dependence of the latter on them, reduce the external risk of the spillover of cutting-edge scientific knowledge, enhance the uniqueness of scientific knowledge, and increase the enthusiasm for pharmaceutical firms' R&D investment (13). The second hypothesis is proposed based on the above analysis:

H2: In the scientific collaboration network, the pharmaceutical firms' structural holes have positive effects on their technological innovation performance.

### Moderators of network positions and pharmaceutical firms' technological innovation performance link

In the social network analysis, network position indicators focus on nodes in the network, but the strength of ties in the network is not involved. However, previous studies have pointed out that strong ties have a positive impact on technological innovation due to the trust mechanism (6, 8), which can reduce coordination costs in scientific collaboration. Therefore, we propose that the strength of scientific collaboration may affect the link between degree centrality and technological innovation performance. In addition, there is an ancient paradox that has always plagued managers of new products according to the knowledge-based view, that is, how to take advantage of core capacities without being hampered by their dysfunctional pip side (32). Patent stock is the embodiment of the core capabilities of pharmaceutical firms. Meanwhile, structural holes help pharmaceutical firms to gain heterogeneous resources and information. We wonder whether the combination of patent stock and structural holes will be beneficial to the technological innovation of pharmaceutical firms. Thus, our study treats the strength of scientific collaboration and patent stock as moderators of network positions and pharmaceutical firms' technological innovation performance link.

### The moderating effects of scientific collaboration strength

The strength of scientific collaboration reflects the closeness of a firm's connection to its partners in a scientific collaboration network, which is measured by the time it lasts and the depth of the relationship (8). Sharing knowledge between pharmaceutical firms and URIs can be more difficult than sharing it within organizations, but the strength of scientific collaboration can facilitate knowledge acquisition, especially that of implicit and complex scientific knowledge by building trust with partners (6). Strong ties in scientific collaboration networks also mitigate the uncertainty associated with innovation (6). Scientific collaboration may entail an idea being openly discussed, being rejected, or even not being given due consideration; therefore, pharmaceutical firms open themselves up to the potential risk of knowledge spillover. When strong ties exist, pharmaceutical firms tend to be more accepting of uncertainty surrounding innovation processes and outcomes. The strength of scientific collaboration is advantageous because of the workings of trust (33). Yang et al. (8) pointed out that scientific collaboration network strength positively affected innovation performance. When the scientific collaboration strength of pharmaceutical firms is high, the degree of mutual trust between them and URIs is high, which will reduce the possibility of conflict in collaboration and decrease the cost of coordination. This is especially important for pharmaceutical firms with a high degree centrality. Although pharmaceutical firms with a higher degree centrality have deep knowledge sharing, higher network status, and richer information, there also face higher coordination costs. Therefore, the scientific collaboration strength will wield positive impacts on the link between degree centrality and the performance of technological innovation, maximizing the benefits obtained from the central position of the scientific collaboration network. Strong ties increase the willingness to share knowledge and reduce the costs of finding the right partner, organizing collaboration, and transferring high-quality information and tacit knowledge (23). Therefore, strong ties help pharmaceutical firms with a high degree centrality to improve the scale and quality of scientific knowledge obtained from URIs and jointly promote technological innovation performance. Tortoriello et al. (34) found evidence that tie strength reduced the negative association between cross-unit transfers and knowledge acquisition. Based on the above discussion, the third hypothesis is proposed:

H3: In the scientific collaboration network, scientific collaboration strength positively moderates the inverted *U*-shaped relationship between degree centrality and technological innovation performance.

### The moderating effects of patent stock

**Figure 1:**
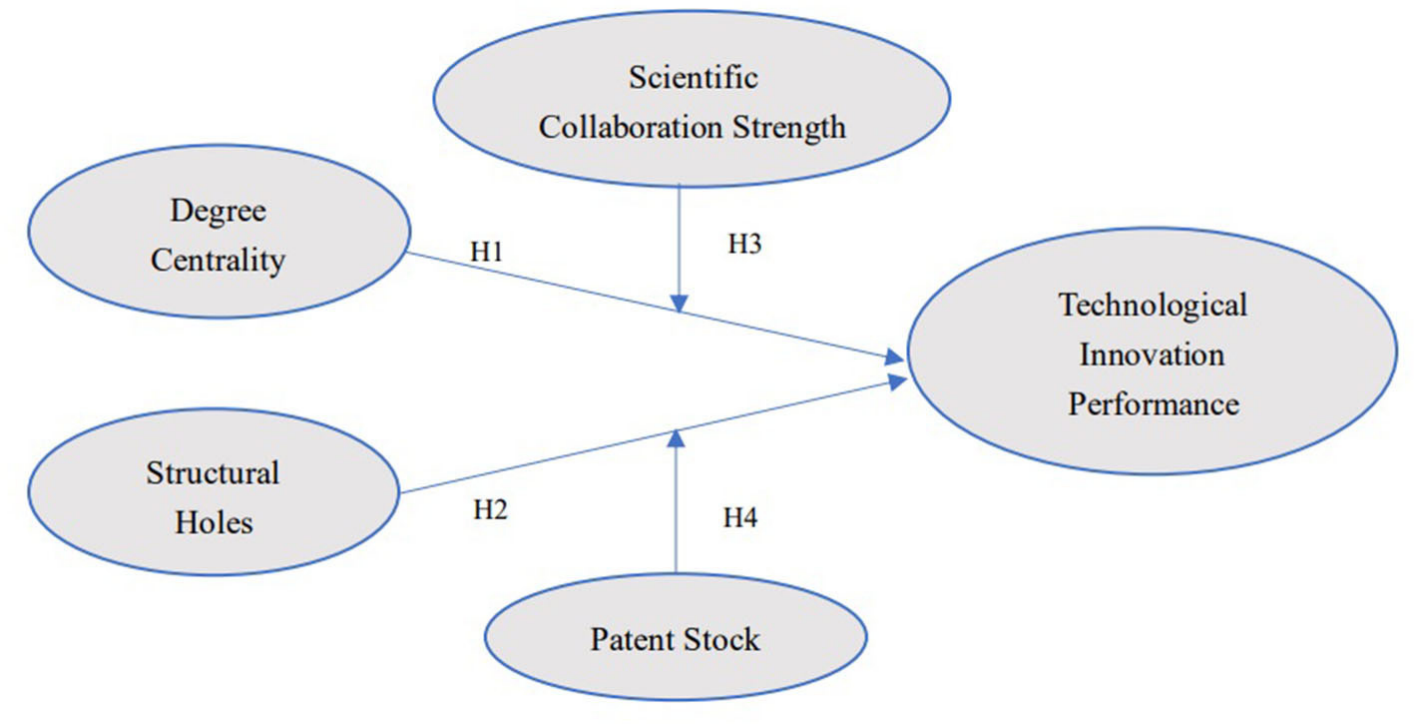
Conceptual framework.

Patents are an important part of a company's proprietary knowledge. Patent stock refers to the number of patents owned by a firm in a certain period (35). The size of a firm's patent stock means the extent to which technical knowledge is available. Patent stock can measure the resources that firms invest in technological innovation, and evaluate the quality and technological capabilities of the firm in specific fields, which provides more options to access and recombine knowledge from URIs in the scientific collaboration network (5). Erden et al. (35) pointed out that the patent stock reflected the reputation of firms in the business community and promoted firms' performance. However, when the number of patents accumulates to a certain level, due to the potential core rigidity and path dependence, the patent stock will reduce firms' innovation output, reflecting the accumulation of non-competitive advantages in the process of technological innovation (36). To better tap into the value of structural holes in the scientific collaboration network for the technological innovation performance of pharmaceutical firms, it is also necessary to ensure that pharmaceutical firms can effectively digest, transform and utilize heterogeneous, implicit, and unique scientific knowledge. That the higher patent stock often means that pharmaceutical firms have a huge technological capacity, so the patent stock will strengthen the impact of structural holes in the scientific collaboration network on the performance of technological innovation. In addition, pharmaceutical firms that occupy more structural holes can obtain different scientific research perspectives and new scientific thinking methods from partners, which can help them solve existing technological innovation problems (37), eliminate the negative impact of path dependence and behavior locking, and accumulate core competitive advantages. Specifically, pharmaceutical firms with large patent stocks can better utilize the advantages brought by the structural holes in scientific collaboration networks, screen favorable external knowledge and information from them, integrate existing technical knowledge with scientific knowledge, give play to the supporting role of scientific knowledge in technological innovation, and ensure better innovation performance. Therefore, the fourth hypothesis is proposed:

H4: In the scientific collaboration network, patent stock positively moderates the relationship between structural holes and technological innovation performance.
  Combining the above hypotheses, the conceptual framework of the study is shown in Figure 1.

## Methodology

### Research setting and data collection

**Figure 2:**
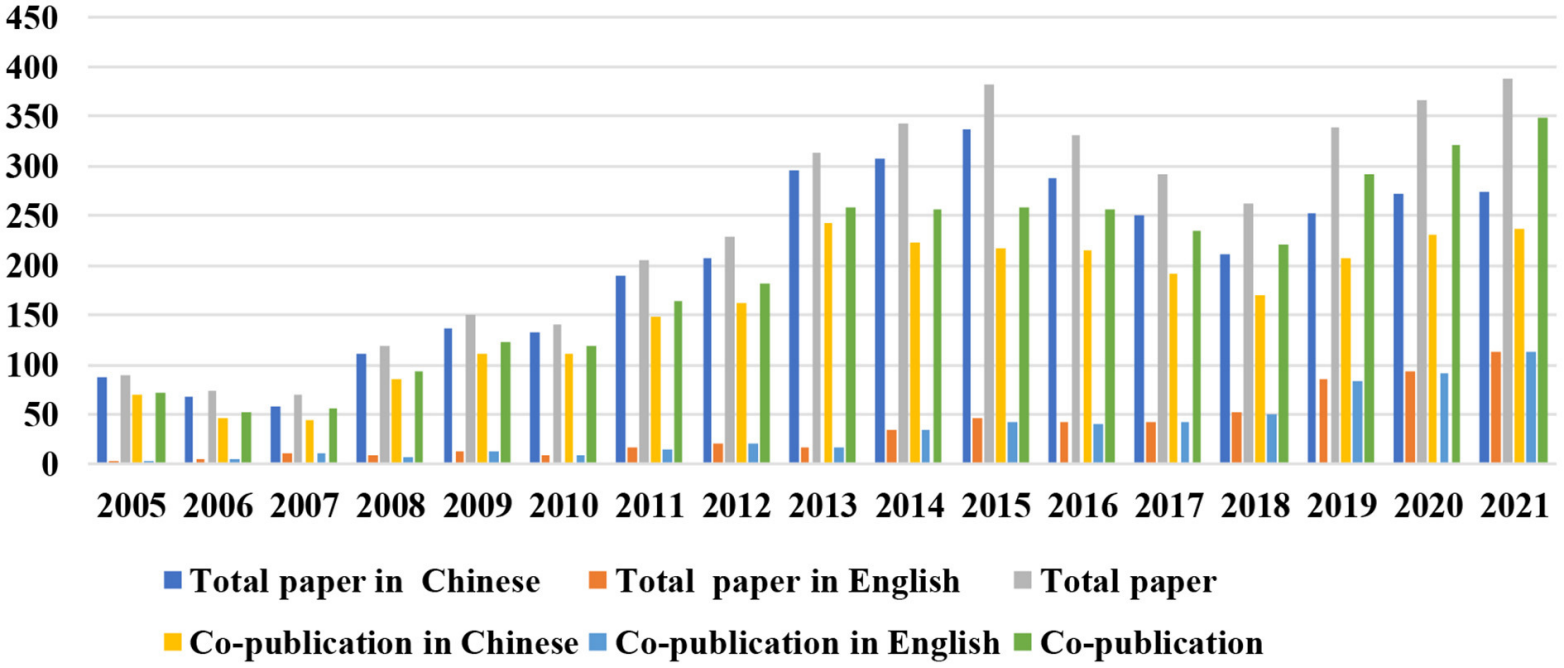
The trend of papers published by Chinese publicly traded pharmaceutical companies during the period 2005–2021.

In this paper, we verify the four hypotheses in the context of Chinese publicly traded pharmaceutical companies based on the consideration of data availability. Data of 301 pharmaceutical companies were downloaded from the CSMAR database from 2007–2021, and 194 companies were obtained as the final research sample after deleting 107 listed companies that belong to the SSE star market, ST, ^*^ST, SST, S. ST listed companies, and publicly traded companies involved in the pharmaceutical industry <2 years. We initially collected basic information and financial indicators of the sample from the CSMAR database. Secondly, co-publication papers in Chinese were collected from the database of “CNKI Academic Journals.” The author's affiliations were the name of sample firms, the search time was 2005–2021, and those papers were indexed by SCI, EI, PKU, CSSCI, and CSCD, which were seen as a sign of better-quality scientific papers in China, and finally, a total of 3,481 Chinese papers were retrieved. Then, English scientific papers were downloaded from the core collection of the Web of Science database. The author's affiliations were searched as names of sample firms, and the search time range was 2005–2021, and a total of 611 English papers were collected. Then, the names of the organizations involved in the paper were carefully proofread, the renamed organizations were merged, and the Chinese and English names of the organizations were examined one by one. Finally, 3,305 co-publishing papers were obtained, involving 1,826 organizations; 2,713 were Chinese papers, and 592 were English papers; the proportion of co-publication in Chinese was 77.94%, and the proportion of co-publication in English was 96.89%. The count results of our data are shown in Figure 2. Furthermore, this paper used 5 years (that is, t-2 to t+2) as the time window to construct 13 scientific cooperation networks following the study of Yang et al. (8). Then we calculated network position indicators of pharmaceutical firms, respectively. Finally, the patent data was obtained from the CNIPA patent search and analysis database in China.

### Measures

#### Dependent variable

Following the previous studies (8, 18), technological innovation performance is measured by the number of innovation patents granted to the firm in years t+1 and t+2 because invention patents are new technical solutions proposed for the method, product, or its improvement, which can better represent the technological innovation level of pharmaceutical firms compared to utility models patents and designs patents in China.

#### Independent variables

Following Yang et al. (8), and Liang and Liu (38), the scientific collaboration network was constructed by co-authorship in publication data, in which five-year moving windows (t-2 to t+2) were applied.

Degree centrality was measured by the number of direct linkages to the focal node following Chen et al. (13) and Martin et al. (39). The specific calculation formula is as follows.


(1) $$\begin{array}{r} C_{degree}\;\left(p_{k}\right)=\underset{i=1}{\overset{n}{\sum }\;}a\;\left(p_{i},p_{k}\right) \end{array}$$

where if the node *p*_i_ is directly connected to the node *p*_*k*_, *a* (*p*_*i*_, *p*_*k*_) = 1, otherwise, *a* (*p*_*i*_, *p*_*k*_) = 0, $\sum_{i=1}^{n}\text{a }\left({\text{p}}_{\text{i}},{\text{p}}_{\text{k}}\right)$is the number of nodes directly connected to the node *p*_*k*_, *n* is the number of nodes in the network.

Structural holes refer to the degree of redundancy in the social network (29). Following Chen et al. (13) and Tortoriello et al. (34), structural holes were calculated using Equation (2).


(2) $$\begin{array}{r} SH_{\text{i}}=1-\underset{j}{\sum \;}{\left(p_{ij}+\underset{q\neq i\neq j}{\sum }\;p_{iq}p_{qj}\right)}^{2} \end{array}$$

where *p*_*ij*_ represents the proportion of relations of node i invested in contacting node j in the network. *p*_*iq*_ represents the proportion of relations of node i invested in contacting node q. *p*_*qj*_ represents the proportion of relations of node q invested in contacting node j. $\underset{\text{q}\neq \text{i}\neq \text{j}}{\sum }\;p_{iq}p_{\text{qj}}$ measures indirect dyadic constraint by considering the strength of third-party ties around dyads i and j. The total in parentheses is the proportion of node i's relations that are indirectly or directly invested in the connection with node j.

#### Moderating variables

Scientific collaboration strength refers to the average number of co-authored papers between focal firms and their partners, which was calculated using Equation (3) following Yang et al. (8).


(3) $$\begin{array}{r} Strength_{\text{it}=}\frac{\underset{t-2}{\overset{{}^{{}^{t+2}}}{\sum }}J_{it}}{\underset{t-2}{\overset{{}^{{}^{t+2}}}{\sum }}K_{it}} \end{array}$$

where *J*_*it*_ represents the number of focal firms' co-publications in the time window (t–2 to t+2), *K*_*it*_ represents the quantity of distinct partners with which the firm coauthored their publications.

Following Erden et al. (35), the patent stock was measured by the number of invention patents granted to firms before the current year (excluding year t).

#### Control variables

Following existing studies (8, 18, 21), we controlled for firm attributes. (a) R&D intensity, measured by dividing R&D expense into sales revenue; (b) Size, measured by the logarithm of total assets; (c) Age, measured by the timespan from the establishment of the firm to the current year; (d) SOE, measured by dummy variables where “1” indicates yes and “0” otherwise; (e) R&D subsidy, measured by the logarithm of the sum of the amount of R&D subsidy plus 1; (f) Export, measured by dummy variables where “1” indicates yes and “0” otherwise; (g) ROA, measured by dividing net profit by average total assets, which refers to return on total assets; (h) Leverage, measured by dividing total debt by total assets. In addition, Year was measured by dummy variables from 2008–2019. The region was measured by dummy variables including areas of Central China, Western China, and Northeast China. The industry was measured by dummy variables including chemical pharmacy and traditional Chinese medicine pharmacy.

### Model specification and estimates

As the dependent variable technological innovation performance was a count variable, which was overdispersion, we used negative binomial regression models to validate our hypotheses. Since negative binomial regression models can be divided into the fixed-effect model and random-effect model using the panel data, we use the negative binomial model with fixed effects according to the results of the Hausman specification test (40).

## Empirical analysis and results

The descriptive statistics and correlations about the main variables in the scientific collaboration network are presented in Table 1. The standard deviations of the dependent variable are greater than its mean; thus, the negative binomial model is appropriate for the research. The mean value of degree centrality is 16.677, and its S.D. value is 47.102, indicating that the degree centrality of pharmaceutical firms in the scientific network is polarizing. Meanwhile, the mean value of structural holes is 0.561, and the value of the standard deviation is 0.321, indicating that structural holes of pharmaceutical firms are more even. As shown in Table 1, the correlation coefficients between the main variables are <0.7, meaning that the discriminant validity is acceptable. The variance inflation factors (VIFs) are all well below the permitted limit of 10, indicating that multicollinearity is not an issue in our model. Moreover, this study standardizes variables including squared terms and interaction terms before regression analysis to avoid the potential multicollinearity issues (41).

**Table 1:** Descriptive statistics and correlations about main variables.

**Variables**	**Mean**	**S.D**.	**VIF**	**1**	**2**	**3**	**4**	**5**	**6**	**7**	**8**	**9**	**10**	**11**	**12**	**13**
1.Technological innovation performance	5.850	11.791	–	1.000												
2.Degree centrality	16.677	47.102	1.35	0.337***	1.000											
3.Structural holes	0.561	0.321	1.32	0.210***	0.262***	1.000										
4.Patent stock	31.63	58.867	1.54	0.334***	0.395***	0.244***	1.000									
5.Scientific collaboration strength	1.023	0.635	1.08	0.072**	0.068**	−0.131***	0.062**	1.000								
6.R&D intensity	5.537	5.049	1.21	0.064**	0.147***	0.028	0.099***	0.063*	1.000							
7.Size	21.766	0.996	2.60	0.115***	0.208***	0.262***	0.354***	−0.022	−0.050**	1.000						
8.Age	17.534	5.609	1.77	−0.100***	0.085***	0.048*	0.074***	−0.065**	−0.020	0.390***	1.000					
9.SOE	0.263	0.441	1.21	−0.011	−0.041	0.061**	−0.049**	0.080***	−0.184***	0.154***	0.052**	1.000				
10.R&D subsidy	16.253	1.416	2.00	0.108***	0.188***	0.243***	0.295***	−0.014	0.098***	0.644***	0.264***	−0.015	1.000			
11.Export	0.517	0.500	1.25	0.016	0.033	0.047*	0.017	0.029	0.024	0.248***	0.108***	0.051**	0.167***	1.000		
12.ROA	0.069	0.072	1.48	0.151***	0.103***	0.115***	0.028	0.048*	−0.053**	−0.009	−0.074***	−0.079***	0.059**	−0.053**	1.000	
13.Leverage	0.309	0.181	1.68	0.007	−0.021	0.056**	0.104***	0.005	−0.090***	0.270***	0.119***	0.265***	0.188***	0.150***	−0.432***	1.000

*p < 0.1, **p < 0.05, ***p < 0.01.

Table 2 represents the results of the regression analysis and explains whether our hypotheses hold. Hypothesis H1 predicts that degree centrality has an inverted U-shaped curvilinear relationship with the technological innovation performance of pharmaceutical firms. As Table 2 shows, the linear term of degree centrality is significantly positive (β = 0.152, *p* < 0.05), while the squared term of degree centrality is significantly negative in Model 2 (β = −0.007, *p* < 0.1). Hence, the result provides strong support for H1. The regression result of structural holes is significantly positive in Model 3 (β = 0.095, *p* < 0.05), indicating that structural holes have a positive impact on firms' technological innovation performance, thus, Hypothesis H2 is proved.

**Table 2:** The negative binomial regression for technological innovation performance.

	**Model 1**	**Model 2**	**Model 3**	**Model 4**	**Model 5**	**Model 6**	**Model 7**
R&D intensity	0.009	0.006	0.006	0.005	0.002	0.005	0.002
Size	−0.150*	−0.122	−0.125	−0.131	−0.133	−0.044	−0.034
Age	0.044**	0.073***	0.078***	0.073***	0.080***	0.055**	0.059**
SOE	0.283	0.301	0.311	0.315	0.388*	0.270	0.352
R&D subsidy	−0.013	−0.050	−0.051	−0.054	−0.049	−0.047	−0.044
Export	0.432***	0.359***	0.369***	0.355***	0.271**	0.360***	0.274***
ROA	1.785***	1.515**	1.320**	1.377**	0.900	1.230*	0.823
Leverage	−0.357	−0.118	−0.095	−0.140	−0.216	−0.075	−0.166
Degree centrality		**0.152** ^**^		0.136*	0.165*	0.177**	0.228**
Degree centrality squared		**−0.007** ^*^		−0.006	−0.020**	−0.007*	−0.029***
Structural holes			**0.095** ^**^	0.088*	0.055	0.079*	0.040
Scientific collaboration strength					0.012		0.053
Degree centrality × scientific collaboration strength					**0.290** ^**^		0.361**
Degree centrality squared × Scientific collaboration strength					**-0.033** ^*^		−0.047**
Patent stock						−0.414***	−0.477***
Structural holes × Patent stock						**0.177** ^**^	0.234***
Constant	3.514**	3.352*	3.360*	3.606**	3.811**	1.829	1.962
Industry	Yes	Yes	Yes	Yes	Yes	Yes	Yes
Year	Yes	Yes	Yes	Yes	Yes	Yes	Yes
Region	Yes	Yes	Yes	Yes	Yes	Yes	Yes
No. of firms	160	135	135	135	130	135	130
No. of observation	1,161	924	924	924	881	924	881
Log likelihood	−2, 067.830	−1, 743.275	−1, 742.851	−1, 741.334	−1, 646.798	−1, 729.053	−1, 632.492
Prob > chi^2^	0.000	0.000	0.000	0.000	0.000	0.000	0.000

*p < 0.1, **p < 0.05, ***p < 0.01. Due to missing data, the number of firms differs across models.

**Figure 3:**
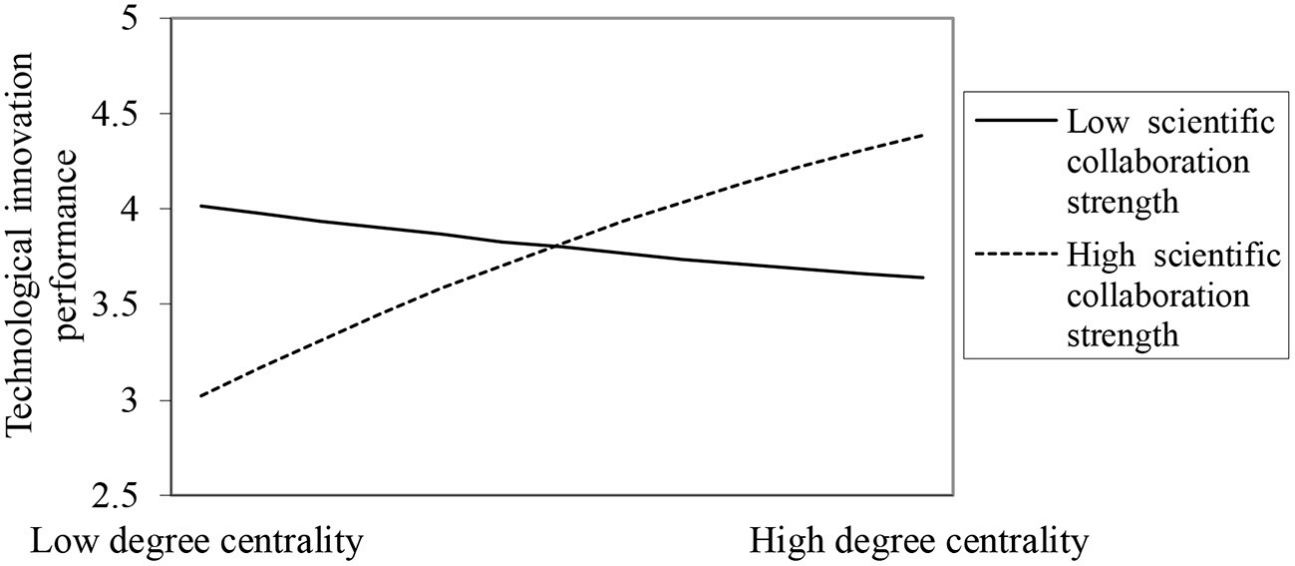
The moderating effects of scientific collaboration strength.

Hypothesis H3 predicts that scientific collaboration strength positively moderates the inverted *U*-shaped relationship between degree centrality and technological innovation performance. As shown in Table 2, the coefficient of Degree centrality^*^ Scientific collaboration strength is positive and significant (β = 0.290, *p* < 0.05), and the coefficient of Degree centrality squared^*^ Scientific collaboration strength is significantly negative in Model 5 (β = −0.033, *p* < 0.1), thus the results support H3. The result is plotted in Figure 3 to clearly illustrate the moderating effect of scientific collaboration strength on the relationship between degree centrality and technological innovation performance. We can see that when scientific collaboration strength is high, the positive slope of degree centrality on technological innovation performance is larger, indicating that scientific collaboration strength enhances the positive effect of degree centrality on technological innovation performance, which supports H3. As shown in Figure 3, the interesting finding is noteworthy: the degree centrality negatively influences technological innovation performance when scientific collaboration strength is low. This indirectly confirms that occupying a higher degree centrality in the scientific collaboration network indicates higher costs.

**Figure 4:**
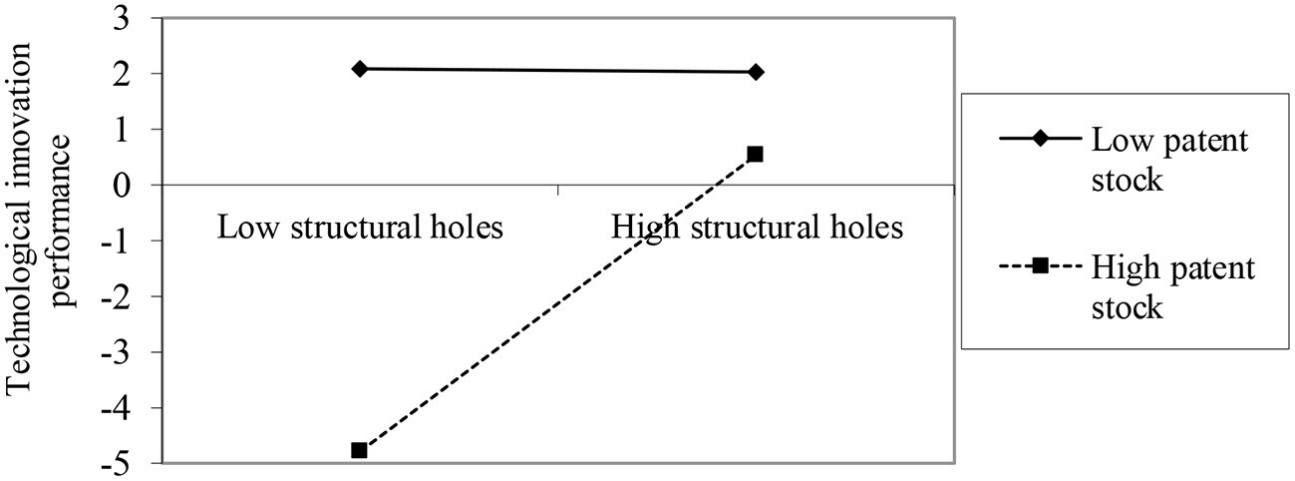
The moderating effects of patent stock.

Hypothesis H4 predicts that patent stock enhances the positive association between structural holes and technological innovation performance. As shown in Table 2, the coefficient of Structural holes ^*^ Patent stock is positive and significant in Model 6 (β = 0.177, *p* < 0.05), hence, the results support H4. The result is plotted in Figure 4 to clearly illustrate the moderating effect of patent stock on the association between structural holes and technological innovation performance. We find that when structural holes are high, the positive slope of structural holes on technological innovation performance is larger, indicating that the positive effect of structural holes on technological innovation performance will be strengthened by patent stock, which supports H4. As shown in Figure 4, when patent stock is low, firms with lower patent stocks have higher innovation performance compared with those with larger patent stocks. The findings of this study are in line with the research of Roper and Hewitt-Dundas (36), indicating the core rigidity of the accumulation of technical knowledge. But our findings go a step further and show that more structural holes in scientific collaborative networks can reverse the negative effects of core rigidity.

We conducted several additional robustness checks. First, considering simultaneous effects of independent variables and moderating variables, we introduce network positions together in Model 4 and incorporate all variables into regression analysis in Model 7. The result shows that the regression results are reliable. In addition, the Hausman test in our study demonstrates that the negative binomial model with the fixed effect is more appropriate than the model with the random effect for our study. To guarantee robustness, we estimate models with random effects, and we notice that the regression results are robust. Third, using innovation patents granted in *t* year as research outputs, we do a negative regression analysis and discover that the regression results are reliable. Fourth, utilizing lag network positions and moderating variables as research inputs, we analyze negative regression results and find that the regression findings are also robust. In conclusion, our results have a satisfactory level of reliability.

## Discussion

### Research findings and discussions

The objective of this study is to investigate whether and how Chinese pharmaceutical firms' positions in the scientific collaboration network promote their technological innovation performance. For this purpose, we construct 13 inter-organizational scientific collaboration networks of 194 publicly traded pharmaceutical enterprises in China. We discover that the network positions in scientific collaboration networks of pharmaceutical companies have a considerable impact on their technological innovation performance. Specifically, degree centrality has an inverted *U*-shaped relationship with technological innovation performance, structural holes are positively associated with it. These findings extend the empirical research on the impact of inter-organizational scientific collaboration on pharmaceutical firms' technological innovation performance from a network perspective. Most of the existing literature ignores this research perspective. Moreover, we investigate the contingent roles of scientific collaboration strength and patent stock on the association between network positions in scientific collaboration and technological innovation. Scientific collaboration strength reinforces the positive effect of degree centrality, which conforms to the extant studies that have proven a positive effect of scientific collaboration strength on firm innovation (8). We expand on it and find that when scientific collaboration strength is higher, firms will benefit from a high degree centrality in the scientific collaboration. Patent stock is negatively associated with firm technological innovation, which shows no difference from previous studies (36). However, we prove that when patent stock is high, firms will take advantage of high structural holes in the scientific collaboration network, which develops the empirical studies concerning the core rigidity of the accumulation of technical knowledge.

### Implications for theory

This paper makes contributions to the academic debates on scientific collaborations. Even though numerous researchers supported that scientific collaboration is conducive to firm innovation (8, 10), some contended that scientific cooperation did not necessarily facilitate innovation (2, 11). In this paper, we explore whether firms gain from scientific collaboration from a social network viewpoint, and discover that the effects of scientific collaboration on firms' technological innovation performance are impacted by their network positions.

The study also demonstrates the contingent value of scientific collaboration strength and patent stock. Although previous literature has perceived the potential positive effects of strong ties on firm innovation (8), our paper shows that the effects of degree centrality will depend on the strength of scientific collaboration. Though the negative effect of patent stock on technological innovation also has been discussed in previous literature (36), this study proves that the effects of structural holes will be moderated by patent stock. As a result, our paper provides nuances to the existing study on the effects boundaries of different network positions in scientific collaboration.

As Yang et al. (8) noted, most of the prior studies of firm scientific collaboration are based on joint patent applications, and co-authorship networks have not been fully explored. This study makes contributions to the research on the association between scientific research and technological innovation, which is one of the earliest studies concerning the relationship between science and technology in the research field of pharmaceutical firms (4).

We also extend the research on technological innovation of pharmaceutical firms in the newly industrialized economy context. As discussed earlier, the technological innovation of pharmaceutical firms in the newly industrialized economy differs from that of the developed countries (2). However, little research has been devoted to the technological innovation of pharmaceutical firms in China from a social network perspective so far. In our paper, the association between scientific collaboration and technological innovation of Chinese pharmaceutical firms is investigated systematically.

### Implications for practice

For firm administrators, our study suggests that enterprises should build a scientific collaboration network and occupy either medium degree centrality or high structural holes to enhance their technological innovation performance. In addition, firms also should be wary of the negative impact of excessive-high degree centrality in scientific collaboration. This study also finds contingent values of scientific collaboration strength and patent stock, which suggests that firms should adjust their network positions in scientific collaboration networks based on their internal conditions. Specifically, when the scientific collaboration strength is high, firms should collaborate with more URI partners to get a high degree centrality in the scientific collaboration network. Given the negative effect of core rigidity in technological knowledge stock, firms should occupy more structural holes to get more heterogeneous scientific knowledge when the patent stock is high.

For government policymakers, this paper argues that they should encourage pharmaceutical firms to research with URIs and occupy key network positions. Meanwhile, governments should urge firms to collaborate with URIs repeatedly and build a relationship of mutual trust, which may better fit a broad scientific collaboration network. In addition, the government may adjust the assessment criteria for pharmaceutical firms so that they will be rewarded for conducting not only patent applications but also related academic paper publications. Given the increasing uncertainty in public health during the COVID-19 period, public policies ought to play more important roles in improving the technological innovation performance of pharmaceutical firms.

### Limitations and future research

Although this study investigates the impact and boundaries of positions in the scientific collaboration network of pharmaceutical firms systematically, it has some limitations. First, as Yang et al. (8) noted, co-authored publications have limitations in the field of measuring scientific collaboration. For instance, “hidden” scientific collaborations cannot be captured, such as sharing facilities and data in private and exchanging ideas through meetings. Besides, we focused on papers co-published in CNKI and Web of Science, but academic books and basic research reports are not included, which are also important output information (13). Therefore, it is suggested that future research explore the impact of network positions by integrating publication papers with other sources of data. Second, we only studied two network positions in scientific collaboration networks: degree centrality and structural holes. Other important dimensions, e.g., different brokerage roles and cliques, should be investigated in future studies. In addition, this study only considered the moderating role of scientific collaboration strength and patent stock when exploring the influence boundary of different network positions on technological innovation performance. Future studies can probe other important moderators, such as R&D incentive policies, digital transformation, etc., to get promising and richer research results. Third, following previous studies, this paper selected the number of invention patents granted to firms to measure their technological innovation performance, but patents are only one of the external indicators of technological innovation performance. Future studies can measure it by other important indicators, e.g., sales of new products and the number of new drugs. Finally, this empirical study is limited to publicly traded pharmaceutical companies in China. However, the methods in this paper can be replicated. Future studies can validate our research framework using data from unlisted pharmaceutical firms or pharmaceutical firms in other emerging countries.

## Data availability statement

The original contributions presented in the study are included in the article/Supplementary material, further inquiries can be directed to the corresponding author.

## Author contributions

X-XW: design of the research, raw data collation, data analysis and results interpretation, article writing, and revision. H-YJ: part of the raw data collation and article revision. All authors contributed to the article and approved the submitted version.

## Funding

This work was supported by the Social Science Project of the Education Department of Jilin Province, China (Grant No. JJKH20200596SK) and the Jilin Provincial Key Laboratory of Human Health Status Identification and Function Enhancement, China (Grant No. 20200601004JC). In addition, we thank X. L. Zhong for her fruitful assistance.

## Conflict of interest

The authors declare that the research was conducted in the absence of any commercial or financial relationships that could be construed as a potential conflict of interest.

## Publisher's note

All claims expressed in this article are solely those of the authors and do not necessarily represent those of their affiliated organizations, or those of the publisher, the editors and the reviewers. Any product that may be evaluated in this article, or claim that may be made by its manufacturer, is not guaranteed or endorsed by the publisher.
